# Reasoning in Reference Games: Individual- vs. Population-Level Probabilistic Modeling

**DOI:** 10.1371/journal.pone.0154854

**Published:** 2016-05-05

**Authors:** Michael Franke, Judith Degen

**Affiliations:** 1 Department of Linguistics, University of Tübingen, Wilhelmstrasse 19, 72072 Tübingen, Germany; 2 Department of Psychology, Stanford University, 450 Serra Mall, Stanford, CA 94305, United States of America; University of Akron, UNITED STATES

## Abstract

Recent advances in probabilistic pragmatics have achieved considerable success in modeling speakers’ and listeners’ pragmatic reasoning as probabilistic inference. However, these models are usually applied to population-level data, and so implicitly suggest a homogeneous population without individual differences. Here we investigate potential individual differences in Theory-of-Mind related depth of pragmatic reasoning in so-called *reference games* that require drawing ad hoc Quantity implicatures of varying complexity. We show by Bayesian model comparison that a model that assumes a heterogenous population is a better predictor of our data, especially for comprehension. We discuss the implications for the treatment of individual differences in probabilistic models of language use.

## Introduction

When Jones complains “I hurt my finger” we are inclined to believe that he is not referring to his thumb, at least much more than when Smith complains “I hurt my toe” we are inclined to believe that he is not referring to his big toe [1, 2]. That a particular mention of “finger” is understood as “a finger other than a thumb” is not a matter of semantic meaning, because most humans have ten fingers and no additional thumbs. Instead, the pragmatic inference that Jones is is not referring to his thumb arises, according to standard pragmatic theory, because the word *thumb* is a short and salient alternative expression that Jones would likely have used if indeed he meant to refer to his thumb, because that would have been an easy and natural way to increase the information content of his utterance. In contrast, the expression *big toe* is not equally readily at hand for Smith, and so the pragmatic inference that Smith has not hurt his big toe is hampered, if it goes through at all.

The influential approach of philosopher Paul Grice [3] tries to explain pragmatic reasoning patterns like the above as the concomitant of regularities of language use, which in turn he described in terms of certain rules of conduct for cooperative speakers, the so-called Maxims of Conversation. One of these is the Maxim of Quantity, which requires, roughly put, that speakers be maximally (but not redundantly) informative given the current purpose of conversation. Pragmatic inferences, amongst them so-called Quantity implicatures like the inference from “finger” to “not thumb,” can be explained by the assumption that listeners believe that speakers (by and large) behave in accordance with Grice’s speaker rules. Modern linguists and psychologists like to preserve Grice’s main ideas, while acknowledging that pragmatics is an uncertain, non-deterministic affair: computing a speaker’s intended meaning requires integrating multiple contextual cues in a limited amount of time, with possibly a substantial amount of uncertainty about relevant contextual parameters like the speaker’s knowledge state or her preferences over and awareness of linguistic alternatives [4]. The resulting picture is that inference patterns like Quantity implicatures are typically observed in the population as relatively robust, but probabilistic trends [5–12].

*Probabilistic pragmatics* is a recent attempt to combine the Gricean approach with the desire to explain complex and contextually-variable empirical data [4]. Probabilistic models have been given for a variety of phenomena, including reasoning about referring expressions [10, 12–15], knowledge implicatures [7], non-literal interpretation [16, 17], vague gradable adjectives [18, 19], syllogistic reasoning [20], or the use of quantifiers [21]. Though different in detail, these models share key ideas. For one, most models include probabilistic versions of Gricean speakers and Gricean listeners. For another, model predictions are usually assessed based on *population-level data* from suitable experimental tasks. That is, the data to be explained by a given model are obtained by averaging over the answers of all participants.

Here, we would like to extend probabilistic modeling of pragmatic language use to acknowledge potential individual-level differences. One general reason for doing so is that it is well known that what best describes a population’s average behavior need not necessarily be a good description of the behavior of the individuals that comprise the population [22–24]. Another reason specific to pragmatics is that there is evidence in the psycholinguistic literature that listeners track speaker-specific (i.e., individual-level) features of their interlocutors, including pragmatic features like the propensity towards over- or under-informativeness [25, 26]. In order to bring probabilistic pragmatics closer towards modeling real speaker/listener behavior, this type of evidence suggests that it is vital to incorporate the possibility of individual differences. Moreover, experimental results indicate restrictions on the depth of Theory-of-Mind (ToM) reasoning capacities in strategic situations (reasoning about the beliefs of agent *i* about the beliefs of agent *j*…) [27–29]. Probabilistic pragmatics models typically assume that Gricean speakers are level-1 ToM-reasoners, in the sense that speaker models consider listeners to be literal interpreters who do not themselves reason about the speaker’s behavior, beliefs or desires. Similarly, Gricean listeners are assumed to be level-2 ToM-reasoners, in the sense that they consider speakers to be the aforementioned level-1 ToM-reasoners. But since like-minded game-theoretic models also consider other possible reasoning types [30–33], it becomes an empirical question which of these types are credible in the light of experimental data, and a technical challenge how to design a formal model of probabilistic pragmatic reasoning that can accommodate potential individual-level differences.

In order to address these issues, we take a data-oriented approach that infers (probabilistically) a language user’s likely reasoning depth from their empirically observed behavior. We formulate models of different complexity: one that assumes a homogeneous population of Gricean speakers and listeners, and one that assumes a heterogeneous population with varying proportions of pragmatic reasoning types inspired by game-theoretic approaches. We assess these models, using Bayesian model comparison [34–36], based on experimental data.

The motivation for this Bayesian approach is that it naturally weighs a model’s complexity in determining its quality. This is particularly important for our case, since we are comparing a simpler model (homogeneous population) to a more complex one (heterogeneous population) with equal numbers of free parameters (see below). The complex heterogeneous model can accommodate every potential data point at least as well as the simple homogeneous model, because the latter is a special case of the former. To compare models, we therefore need to look at *predictive adequacy*, i.e., a model’s ability to predict the actually observed data before having seen it. This way, the complex heterogeneous model is only favored over the simpler homogeneous model if individual differences attested in the data are sufficiently surprising under the simple model from a predictive point of view, where what counts as sufficient surprise is a function of relative model complexity.

Data come from repeated measures *reference games* (to be introduced presently). These tasks have inspired the probabilistic pragmatics model [10] that most recent work builds on. Conclusions based on data from reference games do not necessarily generalize to other areas of language use; rather our study provides the necessary starting point for extended probabilistic modeling of individual differences in more complex and perhaps more natural situations.

## Reference games & pragmatic reasoning types

**Fig 1:**
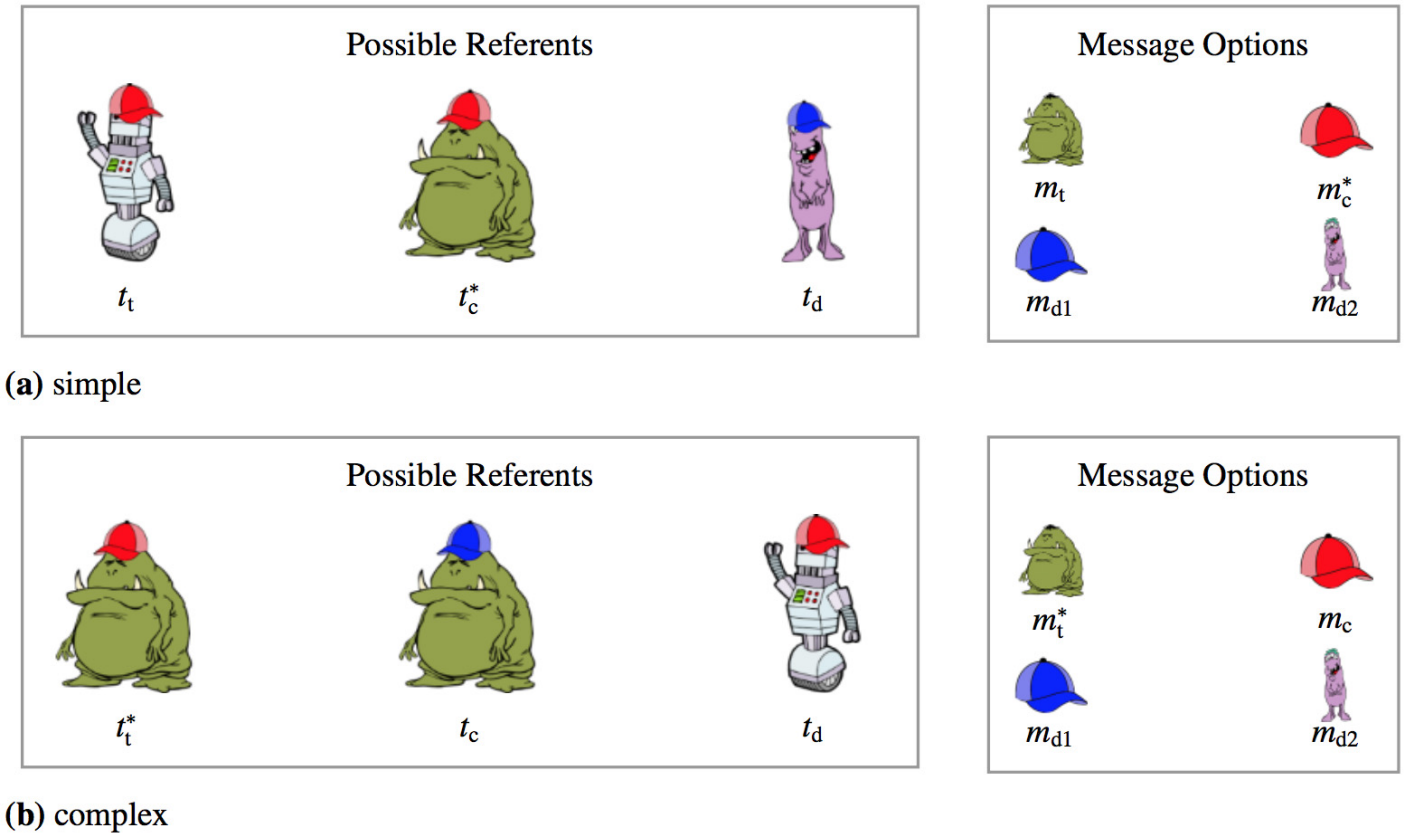
Example contexts that require pragmatic reasoning of varying complexity.

Reference games are an experimental tool for studying the nature and depth of pragmatic reasoning in a controlled laboratory setting [9–15, 37]. Relevant examples are in Fig 1. There are three referents and four possible messages, which are assumed to be common ground between speaker and listener. The speaker’s task is to describe the *trigger referent* (marked by an asterisk) with one of the *message options* from set *M* = {*m*_t_, *m*_c_, *m*_d1_, *m*_d2_}. The listener’s task is to guess the speaker’s intended referent from the set of *possible referents* from set *T* = {*t*_t_, *t*_c_, *t*_d_}, after receiving the trigger message (marked by an asterisk). Indices on referents and messages stand for *t*arget, *c*ompetitor and *d*istractor choices for reasons that will become clear presently.

### Gricean reasoning

How would idealized Gricean language users behave in reference games? Gricean speakers would choose the most informative description, while Gricean listeners would infer the intended referent by assuming that the speaker is Gricean. Consider the case from Fig 1a. The trigger referent is $t_{\text{c}}^{*}$, the green monster with a red hat. To describe this referent, there are two true descriptions: the target message *m*_t_ “green monster” and the competitor message *m*_c_ “red hat.” The other messages are distractor messages that are not true of the trigger referent. A Gricean speaker should choose the target message *m*_t_, because it is more informative than the competitor message *m*_c_: there is only one green monster in the context, while there are two objects with red hats. Turning to comprehension, there are two referents of which the trigger message $m_{\text{c}}^{*}$ (“red hat”) is true: the target referent *t*_t_ (robot with red hat) and the competitor referent *t*_c_ (green monster with red hat). A Gricean speaker would describe the latter as “green monster,” but there is no true message other than the trigger message to describe the target referent. Hence, a Gricean listener would choose the target referent *t*_t_ as the interpretation of the trigger message $m_{\text{c}}^{*}$.

The complexity of reasoning necessary to rationalize the choice of a target referent or target message can vary. In some cases, Gricean reasoning may not be enough, in other cases it may be that simpler reasoning patterns do the trick as well. To see this, and to define other plausible reasoning types with a clear measure of ToM-reasoning complexity, it helps to construe reference games as signaling games.

### Reference games as signaling games

A reference game is an instantiation of a *signaling game* [38], albeit one in which signals already have a conventionally recognized meaning [39–41]. Making this connection is important because it allows us to relate the recent literature on reference games with a rich tradition on pragmatic reasoning in signaling games [32, 33, 42–48].

A signaling game is a game between two players, often called *sender* and *receiver*. The sender is our speaker; the receiver our listener. A signaling game consists of a set of states *T*, drawn from a prior distribution Pr ∈ Δ(*T*). The sender knows the actual state, but the receiver does not. The sender can choose a message from a commonly known set *M*. We assume that messages have conventional meanings, so that 〚*m*〛 ⊆ *T* is the denotation (a subset of states/referents) of message *m* ∈ *M*. The receiver observes this signal and chooses an act from set *A*. Often, the signaling game is conceived as an *interpretation game* in which the receiver’s acts are identified with the states: *A* = *T*. If the actual state is *t* and the receiver’s chosen interpretation is *t*′, then the utility for both sender and receiver is U(*t*, *t*′) = 1 if *t* = *t*′ and 0 otherwise.

### Reasoning types

Pragmatic reasoning in signaling games can be formalized in several ways. One approach relevant for our purposes is *iterated best response reasoning* [30–33] that is essentially an application of like-minded approaches from behavioral economics to linguistics [27, 49–52]. There are several versions of iterated best response reasoning (see [53] for overview and comparison), but a central idea is that there is a hierarchy of reasoning types: starting with literal language users, each higher-level type behaves rationally in response to lower-level types. Concretely, assume that at level 0 we have speakers and listeners who just speak and interpret literally. A level-0 sender (*S*_0_) uses only true signals, and every true signal is equally likely to be chosen. A level-0 receiver (*R*_0_) interprets every signal literally, and every state in which the signal is true is considered an equally likely interpretation. Level-(*n* + 1) language users choose optimal signals or interpretations based on the belief that the other player is a level-*n* player.

A level-1 sender (*S*_1_) is a Gricean speaker, a maximizer of relevant information: on the assumption that the listener selects every true interpretation with equal probability, an *S*_1_ speaker prefers an utterance of *m* over that of *m*′ to express state *t* if *m* is true in *t* and 〚*m*〛 ⊆ 〚*m*′〛. A level-2 receiver (*R*_2_) is then a Gricean listener who assumes that the speaker is a Gricean *S*_1_. But other player types are plausible as well. For instance, the interpretation behavior of a level-1 receiver (*R*_1_) closely aligns with the predictions of *exhaustive interpretation* (see [32]), which is a certain formalization of pragmatic inference that is popular in theoretical linguistics (e.g., [54–58]). A level-2 sender (*S*_2_) is a hyper-pragmatic speaker who chooses the most informative true utterance based on the assumption that the listener is an exhaustive *R*_1_ listener.

Given the richness of reasoning types considered in the theoretical literature, the empirical question arises which of these reasoning types are good predictors of participants’ behavior in reference games. The simple and complex reference games from Fig 1 help address this issue, because different reasoning types are predicted to make different choices in these games. (See S1 Text for a derivation of these predictions.) Table 1 summarizes the reasoning types that we consider here, their level of Theory-of-Mind reasoning and the choices these idealized types would make in the simple and complex games from Fig 1.

**Table 1:** Idealized pragmatic reasoning types and choices in the simple and complex reference games from Fig 1.

	type		choice	
level	speaker	listener	simple	complex
0	literal	literal	target or competitor	target or competitor
1	Gricean	exhaustive	target	target or competitor
2	hyper-pragmatic	Gricean	target	target

## Probabilistic reasoning types

Our goal is to infer, based on empirical data, which pragmatic reasoning types are plausible. In order to allow for slack, errors and mistakes when we fit a reasoning type model to potentially noisy empirical data, we formulate probabilistic variants of the idealized types from Table 1. We then compare a homogeneous “null-model” that contains only the Gricean types to a “saturated model” that contains all of the archetypes from Table 1.

### Homogeneous model

The homogeneous “null-model” is a version of the influential Rational Speech Act (rsa) model [10]. The rsa model defines parameterized probabilistic versions of a level-1 Gricean speaker and a level-2 Gricean listener. Concretely, the rsa model implements a Gricean speaker with a probabilistic tendency to prefer more informative true descriptions over less informative ones (where the strength of that tendency is a model parameter); and a Gricean listener who interprets expressions by forming a posterior belief, by Bayes’ rule, on the assumption that the speaker behaves in the aforementioned fashion, while also factoring in the salience of objects in a given context (measured empirically; see S4 Text).

The behavior of a hypothetical literal listener *R*_0_ is given by an unbiased choice of a referent of which the received message is true. With U the uniform distribution over *T*:

$$R_{0}\left(t|m\right)=U\left(t|\left\{t^{\prime }|m\;\text{is}\;\text{true}\;\text{of}\;t^{\prime }\right\}\right)\;.$$

rsa’s production rule is a probabilistic approximation to a rational choice of expression, given the belief that the listener interprets literally (as defined by *R*_0_). More concretely, if the intended referent is *t*, the speaker’s utility of sending *m* is log(*R*_0_(*t*∣*m*)), which measures the negative (Kullback-Leibler) distance between *R*_0_’s belief after hearing *m* and the speaker’s degenerate probabilistic belief about the intended referent (i.e., the speaker knows who she wants to refer to). When the speaker has a degenerate belief *P*_*S*_(*t*_k_) = 1 for *t*_k_ the intended referent, then if the listener has belief *P*_*L*_ ∈ Δ(*T*), utility in terms of negative Kullback-Leibler divergence reduces to: $U_{S}\left(P_{S},P_{L}\right)=-\text{KL}\left(P_{S}|P_{L}\right)=-\sum_{i}P_{S}\left(t_{\text{i}}\right)\log\frac{P_{S}\left(t_{\text{i}}\right)}{P_{L}\left(t_{\text{i}}\right)}=-\log\frac{1}{P_{L}\left(t_{\text{k}}\right)}=\log P_{L}\left(t_{\text{k}}\right)$.

The rsa model assumes that the speaker chooses messages with a probability that is proportional to its expected success. This is implemented with a parameterized soft-max choice rule (e.g., [59–61]). The speaker’s expected choice probabilities are:

$$\begin{array}{l} S_{1}\left(m|t;\lambda ,\epsilon \right)\propto S_{1}^{\prime }\left(m|t;\lambda \right)+\epsilon ,\text{ where}\\ \;S_{1}^{\prime }\left(m|t;\lambda \right)\propto \text{exp}\left(\lambda \cdot \left(\text{log}R_{0}\left(t|m\right)\right)\right). \end{array}$$

The parameter *λ* > 0 measures, roughly put, the speaker’s rationality. As *λ* → ∞, the speaker would only make rational decisions, choosing the option that maximizes his expected utility. As *λ* → 0, the speaker chooses any true description with equal probability. The parameter *ϵ* allows a small positive probability for descriptions that are not true of the trigger referent. This, or something like it, is needed in an experimental approach like ours that allows the choice options of participants to deviate from semantic meaning.

Gricean listener behavior is given by Bayes’ rule, based on the salience priors S, which are empirically measured (see S4 Text), and the behavior of a Gricean speaker *S*_1_:

$$R_{2}\left(t|m;\lambda ,\epsilon \right)\propto S\left(t\right)\cdot S_{1}\left(m|t;\lambda ,\epsilon \right)\;.$$

### Heterogeneous model

Taken at face value, the above formulation of a single speaker and a single listener rule, in conjunction with the motivation that this is what a traditional Gricean approach would predict, seems to suggest that *all* speakers and listeners also individually conform to the predictions made by *S*_1_ and *R*_2_. We want to explore the hypothesis that speaker and listener populations are a mix of reasoning types that includes probabilistic variants of all the idealized reasoning types summarized in Table 1. In line with the rsa model we look at the following probabilistic type rules (see S2 Text for motivation and formal details):

$$\begin{array}{l} S_{0}\left(m|t;\lambda \right)\propto \text{exp}\left(\lambda \cdot U\left(m|\{m^{\prime }|m^{\prime }\text{ is true of }t\}\right)\right)\\ R_{0}\left(t|m;\lambda \right)\propto \text{exp}\left(\lambda \cdot U\left(t|\left\{t^{\prime }|m\text{ is true of }t^{\prime }\right\}\right)\right)\\ S_{n+1}\left(m|t;\lambda ,\epsilon \right)\propto S_{n+1}^{\prime }\left(m|t;\lambda \right)+\epsilon \\ \text{ with }S_{n+1}^{\prime }\left(m|t;\lambda \right)\propto \text{exp}\left(\lambda \cdot \text{log}R_{n}\left(m|t;\lambda \to \infty \right)\right)\\ R_{1}\left(t|m,\lambda \right)\propto \text{exp}\left(\lambda \;U_{R_{1}}\left(t,m\right)\right)\;\\ \text{ with }U_{R_{1}}\left(t,m\right)\propto U\left(t\right)\cdot S_{0}\left(m|t;\lambda \to \infty \right)\\ R_{2}\left(t|m;\lambda ,\epsilon \right)\propto S\left(t\right)\cdot S_{1}\left(m|t;\lambda ,\epsilon \right) \end{array}$$

We will assume for modeling convenience that all reasoning types share a *λ* and an *ϵ* (see also the *General discussion* section).

### Nested modeling

The homogeneous model assumes that the population consists exclusively of Gricean types, while the heterogenous model is compatible with any population distribution over the three relevant speaker and listener types *S*_0_, *S*_1_, *S*_2_, *R*_0_, *R*_1_ and *R*_2_. Consequently, the homogeneous model can be conceived of as a special case of the heterogeneous model, in the sense that the former fixes the population distribution to a single value (like a null-hypothesis would; in this case to *S*_1_ and *R*_2_). Since the simpler model is a special case of the complex model, the latter will be able to accommodate every data observation at least as well as the former. On top of that, there are observations that the complex model could accommodate much better than the simpler model. For example, some patterns of behavior in simple or complex reference games, as introduced previously, would seem incompatible with the simpler model, but unproblematic for the complex model (see Table 1). One such pattern would be the observation of exclusively target choices in simple and complex conditions for production (suggesting that all subjects are individually level-2 reasoners), but an equal number of target and competitor choices for comprehension (suggesting level-0 behavior). In order to test whether the more complex, heterogeneous model is necessary or whether the null-model is sufficient, we therefore turn to experimental data from tasks that require the comprehension and production of referential expressions, including drawing Quantity inferences of varying complexity.

## Experiments

Experiment 1 tests the comprehension of referential expressions in reference games like the ones introduced in Fig 1. Experiment 2 tests the production of referential expressions in the same games. Experiment 3 (see S4 Text) elicits salience priors over objects required for modeling the *R*_2_ listener. Links to all experiments are provided in S3 Text.

### Experiment 1: comprehension

Experiment 1 tested participants’ behavior in a comprehension task that used instantiations of the “monsters and robots” reference games from Fig 1.

#### Participants

60 participants were recruited via Amazon’s Web Service Mechanical Turk. Participants’ IP address was limited to US addresses only. Only participants with a past work approval rate of at least 95% were accepted.

#### Ethics statement

This study was conducted with the approval of the Stanford University research subjects review board. All participants gave written consent and received $1.00 for their participation (hourly rate of $10.00) according to the policies set forth by the Stanford University research subjects review board.

#### Procedure

Participants engaged in a referential comprehension task. On each trial they saw three objects on a display. Each object differed systematically along two dimensions: its ontological kind (robot, green monster, purple monster) and accessory (scarf, blue hat, red hat). In addition to these three objects, participants saw a pictorial message that they were told was sent to them by a previous participant whose goal was to get them to pick out one of these three objects. They were told that the previous participant was allowed to send a message expressing only one feature of a given object, and that the messages the participant could send were furthermore restricted to monsters and hats (i.e., there were no messages for referring to the robot or scarf feature; we refer to these features as *inexpressible features*). The four expressible features were visible to participants at the bottom of the display on every trial and are shown on the right side of Fig 1.

Participants initially completed four speaker trials. They saw three objects, one of which was highlighted with a yellow rectangle. Participants were asked to click on one of four pictorial messages to send to another Mechanical Turk worker to get them to pick out the highlighted object. They were told that the other worker did not know which object was highlighted but knew which messages could be sent. The four speaker trials contained three unambiguous and one ambiguous trial which could function as fillers in the main experiment.

#### Materials

Participants saw 66 experimental trials, which were composed of 24 critical and 42 filler trials. Of the 24 critical trials, 12 constituted a simple implicature situation and 12 a complex one (as shown in Section 1). Stimuli were created by randomly sampling a message and then generating a grid of three objects—a target, a competitor, and a distractor— following different constraints in different conditions.

On *simple implicature* trials, the target was generated by combining the feature denoted by the sampled message with the inexpressible feature along the other feature dimension. For example, if the sampled message was one of “red hat” or “blue hat”, the target was a robot with the respective hat. If instead the sampled message was “purple monster” or “green monster”, the target was the respective monster with a scarf. The competitor was generated by combining the feature denoted by the sampled message with a randomly sampled expressible feature along the other feature dimension. For example, if the sampled message was “red hat”, the competitor could be a green monster with a red hat or a purple monster with a red hat. The distractor was generated by combining two features that were randomly sampled from the set of features that did not contain those features already present in target and competitor. For example, if the target was a robot with a red hat and the competitor was a green monster with a red hat, the distractor could be a purple monster with either a scarf or a blue hat.

On *complex implicature* trials, the target was generated by combining the feature denoted by the sampled message with an expressible feature along the other feature dimension. For example, if the sampled message was “green monster” the target could be a green monster with a red hat. The competitor was generated by combining the feature denoted by the sampled message with the remaining expressible feature along the other feature dimension. Continuing our example, the competitor would then be a green monster with a blue hat. The distractor was generated by combining the target feature that was not denoted by the sampled message (red hat) with the inexpressible feature along the other feature dimension (robot).

Of the 42 filler trials, 24 used the displays from the implicature conditions but the target was a) the competitor from the simple condition (six trials), b) the distractor from the simple condition (six trials), or c) the competitor from the complex condition (12 trials), as identified unambiguously by the trigger message. This was also intended to prevent learning associations of display type with the target. On the other 18 filler trials, the target was either entirely unambiguous or entirely ambiguous given the message. That is, there was either only one object with the feature denoted by the trigger message, or there were two identical objects that were equally viable target candidates. *Unambiguous* and *ambiguous* fillers were included as baselines to compare behavior on implicature trials to. Ambiguous fillers establish how often the target could be chosen by chance, while unambiguous fillers establish the upper bound on target choices. We did not include filler items where the target was the distractor from the complex condition, because this would have required participants to draw a one-step inference to identify the target. Trial order as well as target, competitor, and distractor order were randomized.

#### Results and discussion

Those 15% of participants with the highest error rate (distractor responses) on trials that were not ambiguous were excluded from the analysis. This was done to avoid artificially inflating the noise parameter *ϵ* (see section on Bayesian model comparison below for further explanation). The 15% cutoff corresponded to a minimum error rate of 5% and included one participant who was not a self-reported native speaker of English. The data from the 51 remaining participants entered the analysis.

We were interested in participants’ ability to draw simple and complex ad hoc Quantity implicatures. If participants always drew the implicature, their performance on critical trials should pattern with their performance on unambiguous filler trials. If instead they interpreted messages literally, their performance on critical trials should pattern with performance on ambiguous trials.

**Fig 2:**
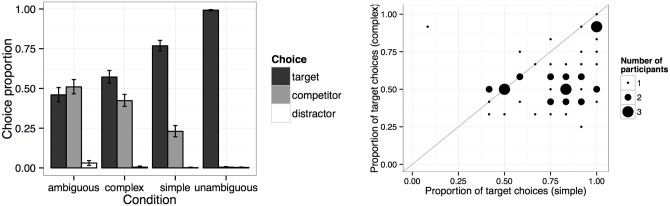
Left: proportions of target, competitor, and distractor choices in Experiment 1. Error bars indicate 95% bootstrapped confidence intervals. Right: proportion of target choices in simple and complex conditions by participant. Dot size indicates number of participants.

Proportions of choice types are displayed in the left panel of Fig 2. As expected, participants were close to ceiling in choosing the target on unambiguous filler trials (99% target choices vs..04% competitor choices) but at chance on ambiguous ones (46% target choices vs. 51% competitor choices). This confirms that participants understood the task. On critical implicature trials, participants’ performance was intermediate between ambiguous and unambiguous filler trials. On simple implicature trials, participants chose the target 77% of the time and the competitor 23% of the time. On complex implicature trials, the target was chosen less often (57% target choices vs. 42% competitor choices).

A logistic mixed-effects regression was conducted to assess whether the observed differences in target choices between the ambiguous and complex, and the complex and simple condition, were significantly different. Unambiguous trials were not included in the analysis because target choices in this condition were at ceiling, i.e. there was not enough variance in participants’ responses to allow the model to converge. Trials on which the distractor was selected were excluded to allow for a binary outcome variable (target vs. competitor choice). This led to an exclusion of 1% of the data.

The model predicted the log odds of choosing a target over a competitor from a Helmert-coded CONDITION predictor. Two Helmert contrasts over the three relevant critical and filler conditions were included in the model, one comparing the simple implicature condition with the other two conditions (Condition (harder vs. simple)), and one comparing the complex implicature to the ambiguous filler condition (Condition (ambiguous vs. complex). This allowed us to capture whether the differences in choice distributions for neighboring conditions suggested by Fig 2 were significant.

We were also interested in whether participants displayed learning effects, i.e., whether they (maybe differentially) improved in the implicature conditions over the course of the experiment. To test this, the model also included a centered trial number predictor and the interaction of trial number and each of the Helmert contrasts. The model also included further control predictors for message type (accessory vs. species, centered) and target position (dummy-coded with left position as reference level). Finally, following [62], the model included the maximal random effect structure that allowed it to converge, which consisted in by-participant intercepts as well as by-participant slopes for message type and trial number.

A model summary is shown in Table 2. As suggested by Fig 2, participants made more target choices in simple than in complex implicature situations (*β* = 1.28, *SE* = .12, *p* < .0001), and they made more target choices in complex implicature situations than on ambiguous filler trials (*β* = .44, *SE* = .13, *p* < .001). This suggests that what we are calling simple implicatures are indeed simpler than what we are calling complex implicatures. Second, it suggests that, while participants performed similarly on complex and ambiguous trials, they nevertheless performed significantly above chance on complex trials. That is, at least some participants computed the more complex two-step implicatures at least sometimes. However, as shown in the right panel of Fig 2, the aggregate 77% (simple) and 57% (complex) target choice pattern was not mirrored on the individual participant level. That is, there was a lot of individual variability in participants’ choice behavior. Some individuals succeeded more often on complex trials than others, and success on complex trials also made it more likely that that individual had a higher success rate on simple trials. This strongly suggests that participants in this experiment were a mixture of *R*_1_ exhaustifiers and *R*_2_ Gricean listeners.

**Table 2:** Summary of coefficients, standard errors, and *p*-values for the comprehension (Experiment 1, left) and production (Experiment 2, right) models. Significant *p*-values are shown in boldface. Predictors coding experimental conditions of interest, predictors coding learning effects, and other control predictors are separated from each other by horizontal lines.

	*Comprehension (Exp. 1)*			*Production (Exp. 2)*		
	Coef *β*	SE(*β*)	p	Coef *β*	SE(*β*)	p
Intercept	−.15	.11	<.18	.55	.13	**<.0001**
Condition (harder vs. simple)	1.28	.12	**<.0001**	1.63	.13	**<.0001**
Condition (ambiguous vs. complex	.44	.13	**<.001**	.20	.11	<.08
Trial	.00	.00	<.3	−.01	.00	**<.05**
Harder.vs.Simple: Trial	.00	.01	<.9	−.01	.01	<.45
Ambiguous.vs.Complex: Trial	.01	.01	<.33	.00	.01	<.49
Target position (middle vs. left)	1.28	.14	**<.0001**			
Target position (right vs. left)	.74	.13	**<.0001**			
Target position				.03	.05	
MessageType	−.02	.12	<.85	.41	.10	**<.0001**

Neither the main effect of trial number nor its interactions with the two Helmert contrasts reached significance, suggesting that there were no learning effects in this study, i.e. participants’ behavior on both implicature and filler trials remained constant throughout. This is important for our subsequent model comparison, which assumes that participants are of a fixed reasoning type.

Finally, both target position effects reached significance: participants were more likely to choose the target if it was in the center (*β* = 1.28, *SE* = .14, *p* < .0001) or right (*β* = .71, *SE* = .13, *p* < .0001) compared to the left position in the display. There was no multicollinearity between fixed effects to speak of (all variance inflation factors < 1.33).

This experiment constitutes a replication of Experiment 1 of [9]. The data from all four conditions will be used in model comparison on the homogeneous and heterogeneous model. Of interest is whether the individual participant variability suggested by Fig 2 is substantial enough to warrant the additional complexity introduced by allowing for heterogeneous types. But first we report the results of the complementary production study.

### Experiment 2: production

Experiment 2 tested participants’ behavior in a production task within the same reference game setting as Experiment 1.

#### Participants

60 participants were recruited via Amazon’s Web Service Mechanical Turk. Participants’ IP address was limited to US addresses only. Only participants with a past work approval rate of at least 95% were accepted.

#### Ethics statement

This study was conducted with the approval of the Stanford University research subjects review board. All participants gave written consent and received $1.00 for their participation (hourly rate of $10.00) according to the policies set forth by the Stanford University research subjects review board.

#### Procedure and Materials

The procedure was identical to that on speaker trials in Experiment 1.

Each participant saw 66 experimental trials. The distribution of trial types was the same as in Experiment 1: 24 critical trials (12 simple, 12 complex, see Fig 1) and 42 filler trials. Each of the four messages received a trial by trial status as target, competitor, distractor 1, or distractor 2. Stimuli were created by randomly sampling a message and then generating a grid of three objects following different constraints in different conditions. The sampled message was the target.

On *simple implicature* trials, the trigger object was generated by combining the feature denoted by the sampled message with an expressible feature along the other feature dimension. The message denoting this other feature was the competitor message. For example, if the sampled message was “green monster”, the trigger object might be a green monster with a red hat. The target message was then “green monster”, the competitor message “red hat”. A second object was generated by combining the feature denoted by the competitor message with the inexpressible feature along the other feature dimension. In our example, the second object would be a robot with a red hat. A third object was generated by combining the two remaining expressible features. The messages denoting these features were randomly deemed *distractor 1* or *distractor 2*.

On *complex implicature* trials, the trigger object was generated by combining the feature denoted by the sampled message with an expressible feature along the other feature dimension. The message denoting this other feature was the competitor message. For example, if the sampled message was “green monster”, the trigger object might be a green monster with a red hat. The target message was then “green monster”, the competitor message “red hat”. A second object was generated by combining the feature denoted by the competitor message with the inexpressible feature along the other feature dimension. In our example, the second object would be a robot with a red hat. A third object was generated by combining the feature denoted by the sampled message with the remaining expressible feature along the other feature dimension. In our example: a green monster with a blue hat. The remaining messages were randomly deemed *distractor 1* or *distractor 2*.

Of the 42 filler trials, 24 used the displays from the implicature conditions but the highlighted trigger object was a) the competitor from the simple condition (six trials), b) the distractor from the simple condition (six trials), or c) the distractor from the complex condition (12 trials). We did not include filler items where the trigger was the competitor from the complex condition, because this would have required participants to draw a one-step inference to select the target message. On the remaining 18 filler trials, the target message to refer to the highlighted object was either entirely unambiguous (because the other feature was one of the inexpressible robot or scarf features) or entirely ambiguous (because no other object in the display had the trigger object features but each trigger object feature was an equally good message choice). *Unambiguous* and *ambiguous* fillers were included as baselines to compare behavior on implicature trials to. On unambiguous trials, the trigger object had one expressible and one inexpressible feature, such that the target message denoted the expressible feature. No other objects in the display shared that feature. On ambiguous trials, the trigger object had two expressible features that no other object in the display shared. Ambiguous fillers establish how often the target message would be chosen by chance, while unambiguous fillers establish the upper bound on target message choices. Trial order, position of the trigger object in the grid, and order of target, competitor, and distractor messages were randomized.

#### Results

Again, the 15% of participants with the highest error rate on trials that were not ambiguous were excluded. This corresponded to a minimum error rate of 10%, which is slightly higher than in Exp. 1 and reflects increased difficulty associated with the production task. For example, participants were asked to choose between four, rather than three, alternatives. The exclusion included two participants who were not self-reported native speakers of English. The data from the 50 remaining participants entered the analysis.

**Fig 3:**
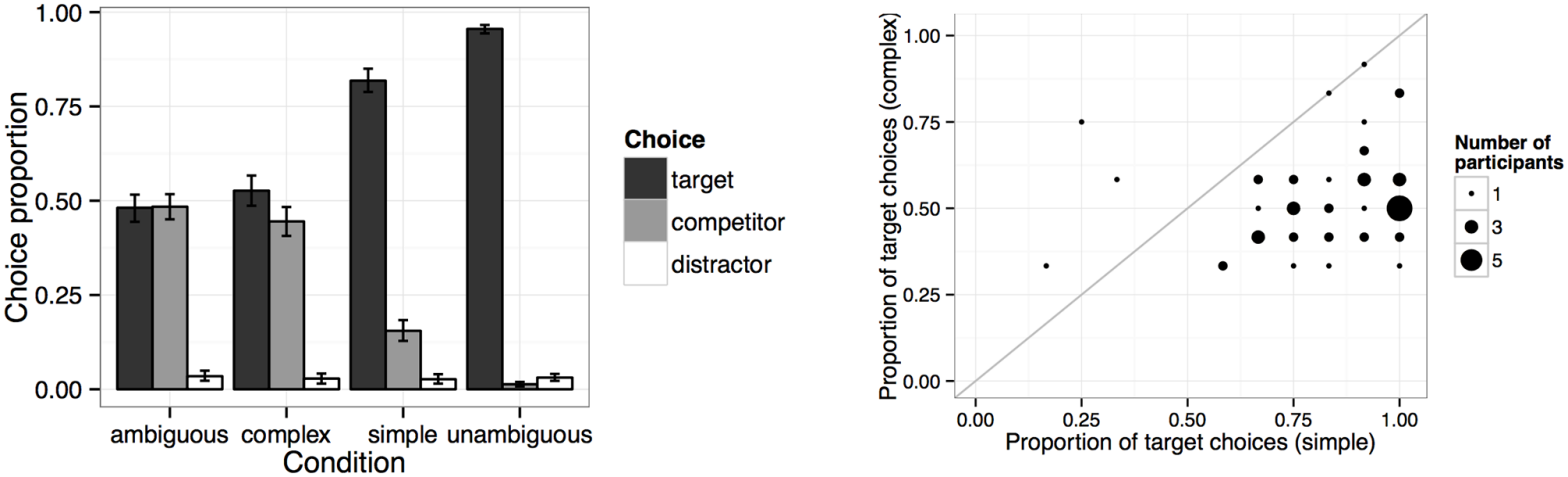
Left: proportions of target, competitor, and distractor choices in Experiment 2. Error bars indicate 95% bootstrapped confidence intervals. Right: proportion of target choices in simple and complex conditions by participant. Dot size indicates number of participants.

Proportions of choice types are displayed in Fig 3 on the left. We collapse the two distractor types into one distractor category, since the difference is of no theoretical interest and there were no differences in the choice distributions between the two categories. As expected, participants were close to ceiling in choosing the target on unambiguous filler trials (96% target choices vs. 1% competitor choices) but at chance on ambiguous ones (48% target choices vs. 48% competitor choices). On critical implicature trials, participants’ performance was intermediate between ambiguous and unambiguous filler trials. On simple implicature trials, participants chose the target 82% of the time and the competitor 16% of the time. On complex implicature trials, the target was chosen less often (53% target choices vs. 45% competitor choices).

The mixed effects logistic regression analysis was conducted as in Experiment 1 and predicted target over competitor message choices. The only differences were that a) the random effects structure consisted only of random by-participant intercepts (because more complex random effects structures did not allow the model to converge) and b) target position was coded as a centered numeric position (1 to 4).

A model summary is shown in Table 2. As suggested by Fig 3, participants made more target choices in simple than in complex implicature situations (*β* = 1.63, *SE* = .13, *p* <.0001). In contrast to the comprehension study, participants only made marginally more target choices in complex implicature situations than on ambiguous filler trials (*β* = .2, *SE* = .11, *p* <.08), suggesting that complex inferences are more difficult to draw in production than in comprehension.

In addition to the theoretical effects of interest, there was a main effect of trial number, such that participants became overall slightly less likely to choose targets as the experiment progressed (*β* = -.01, *SE* = .003, *p* <.05). However, this effect was so small compared to the other significant fixed effects that it is not of interest. Neither interaction of trial number with the two Helmert contrasts reached significance, suggesting that participants’ behavior did not change differentially for the different conditions.

Finally, of the additional control predictors only that of message type reached significance: participants were more likely to choose the target message if the message denoted a species rather than an accessory. There was no multicollinearity between fixed effects to speak of (all variance inflation factors < 1.16).

This experiment constitutes a replication of Experiment 2 of [9], with some caveats: the rank order of target choice proportions is as in [9]. However, here we find that target choices are slightly above chance in the complex condition and target choices are not at ceiling in the simple condition. These differences may be due to the size of the sample used in this experiment, which was larger and thus made it possible to detect smaller effects. The data from all four conditions will be used in the model comparison of the homogeneous and heterogeneous model. As was the case for the comprehension data reported previously, the individual target choice distributions shown in Fig 3 on the right suggest that in production, too, rather than mirroring the aggregate 82% (simple) and 53% (complex) target choice proportions, individual participants displayed systematic differences in response behavior.

## Model fits and model comparison

The main goal of this section is to assess whether it is plausible to maintain the “null hypothesis” that all participants in our experiments can be construed as Gricean, i.e., as *S*_1_ and *R*_2_ respectively. The “alternative hypothesis” is that our data are better captured by assuming that the population of participants is a heterogeneous mix of various reasoning types.

Our conclusion will be that, despite the added complexity of the heterogeneous model, the individual-level comprehension data strongly favor the more complex model. The individual-level production data, on the other hand, suggest that most (though not all) speakers were Gricean in our experiment. For production, there is a large majority of likely Gricean *S*_1_ speakers, but for comprehension there is no majority of Gricean *R*_2_ listeners. What the remainder of this section adds to this is a careful model comparison that weighs predictive accuracy against model complexity. Such model comparison is needed because mere inspection of the choice data alone or inspection of posteriors over reasoning types (see below), will not allow precise assessments: e.g., is it enough evidence to favor the complex model over the simpler model that four participants from our pool are most likely literal speakers?; could the simpler model still be plausibly maintained, given its elegant parsimony, despite the fact that only a minority of subjects are most likely Gricean listeners of level-2? Normatively compelling answers to these questions hinge on the relative complexity of models and on the relative success of explaining the observed data. The Bayesian model comparison of this section offers exactly that.

### Gricean types match the population-level data

**Fig 4:**
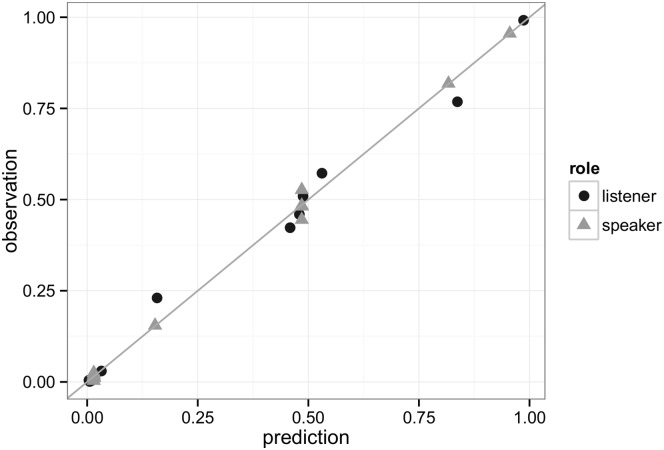
Probabilistic predictions of the rsa model under best-fitting parameter values (see main text), plotted against the observed choice frequencies. Each dot represents one choice option in one of our four reference games.

Before looking at individual-level data, it is worth noting that, like in previous studies on reference games, the Gricean types alone seem to capture the population-level data reasonably well. Abstracting away from salience priors and stochasticity in the choice rules, Gricean speakers *S*_1_ should be able to solve the simple, but not the complex condition, while Gricean listeners *R*_2_ should be able to solve both. The population-level average data, plotted in Figs 2 and 3 on the left, show this only in tendency. But if we add salience and noise, the observed choice frequencies can be approximated rather well by the homogeneous model. Previous studies have looked at point-estimates for parameters like our *λ* and *ϵ*, obtained by minimizing the squared distance between the observed choice frequencies and the predicted choice probabilities [7]. Best fitting parameters in this sense are *λ* = 2.533 and *ϵ* = 0.015 (production) and *λ* = 1.597 and *ϵ* = 0.005 (comprehension). Resulting predictions are well-aligned with the observations, as shown in Fig 4 (correlation *r* = 0.997, *p* < 0.0001). By this standard, the Gricean rsa model appears to be a good predictor of our data at the population-level: on average, speaker and listener behavior appears to be classically Gricean (modulo stochasticity and salience effects).

### A hierarchical model for individual-level data

That a population average is well approximated by some mathematical function does not imply that the individual-level data must conform to it as well [22–24]. It is therefore relevant to look at each individual’s choice data as the to-be-explained observations. A first goal is to infer, based on each individual’s choice data, an estimate of each individual’s likely reasoning type. A second goal is to determine whether the simpler Gricean model is sufficient for explaining the data, or whether the added complexity of the heterogeneous model is necessary. To achieve both goals, we take a hierarchical modeling approach [63] in which the simpler Gricean model is nested under the more complex heterogeneous type model.

The general idea behind this approach is that we specify for each participant *i* a latent type *τ*_*i*_ ∈ {0, 1, 2} that captures the depth of pragmatic reasoning of *i*. Each *τ*_*i*_ is drawn from a *type distribution* *P*^*τ*^ that captures the probability of sampling reasoning types from the population from which our participants were sampled. Our data will then provide information about the likelihood that each participant is a pragmatic reasoner of level 0, 1, or 2 *and* about the general population distribution of reasoning types. The homogeneous model is a special case of the heterogeneous model in that it assumes *a priori* only one value for the type distribution *P*^*τ*^.

More concretely, the data that we would like to explain are counts $d_{ijk}^{X}$, one set for production (*X* = *S*) and one for comprehension (*X* = *R*), giving the number of choices across the whole experiment of participant *i* for game *j* (simple, complex, unambiguous, ambiguous) that fell into category *k* (target, competitor, distractor 1, possibly also distractor 2 for production). Every participant saw each game *j* a fixed number of times $n_{j}^{X}$. We assume that each participant *i* has a fixed type *τ*_*i*_ ∈ {0, 1, 2} that is constant across the experiment. The assumption of temporally consistent reasoning types is generally dubious, but necessary to keep the model manageable. However, in our case this assumption seems warranted given the lack of relevantly significant trial effects in our regression analyses reported above.

For production, a participant’s type fixes whether that participant is a literal *S*_0_, a Gricean *S*_1_ or a hyper-pragmatic *S*_2_ speaker. For comprehension, a participant’s type fixes whether that participant is a literal listener *R*_0_, an exhaustifier *R*_1_ or a Gricean listener *R*_2_. The likelihood of each $d_{ijk}^{X}$ is then given by the binomial distribution, with a probability $P_{ijk}^{X}$ that depends on the participant’s type $\tau_{i}^{X}$, error rate *ϵ*^*X*^, rationality *λ*^*X*^, and game *j*:

$$d_{ijk}^{X}∼\text{Binomial}\left(P_{ijk}^{X},n_{j}^{X}\right)\;P_{ijk}^{X}=X_{\tau_{i}}\left(k|{\text{game}}_{j};\epsilon ,\lambda \right),$$

where the latter is defined by the probabilistic choice types of the heterogeneous model that were introduced previously.

We assume largely uninformative prior probabilities for parameter values (the structure is the same for production and comprehension, so we omit the variable *X* for readability):

$$\begin{array}{l} \epsilon ∼\text{Gamma}\left(\text{shape}=.25,\text{rate}=.1\right)\;\lambda ∼\text{Gamma}\left(\text{shape}=2,\text{rate}=.5\right)\\ \tau_{i}∼\text{Categorical}\left(P^{\tau }\right)\;P^{\tau }∼\text{Dirichlet}\left(1,1,1\right) \end{array}$$

The use of these gamma distributions is motivated by the idea that we expect trembles *ϵ* to be very small, possibly even 0, but that *λ* would be positive, but rather small as well.

**Fig 5:**
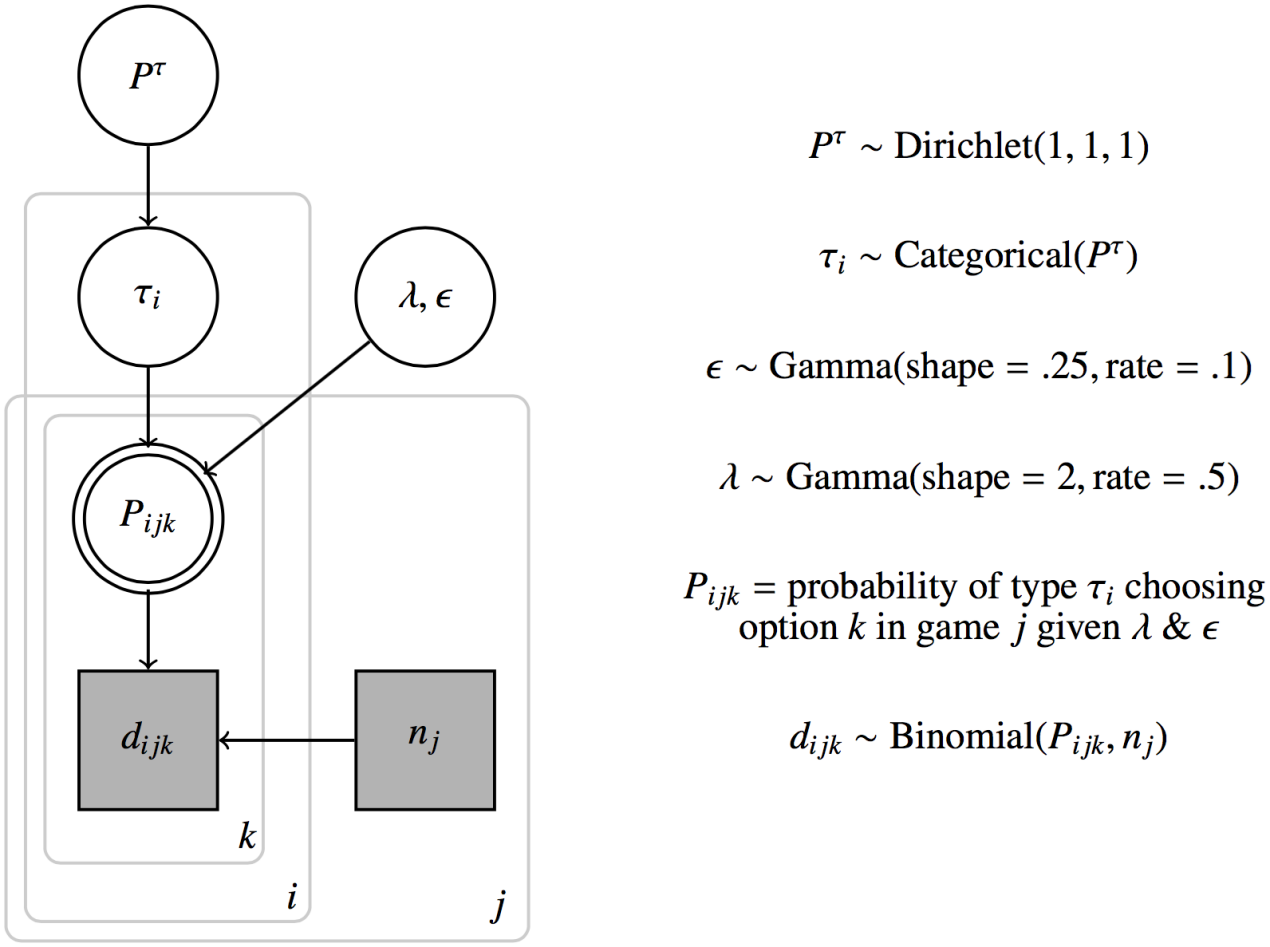
Sketch of the full data-generating model as a probabilistic graphical model, using the conventions of [64]. The more abstract a parameter, the higher it is in the graph. Deterministic variables have double edges. Categorical variables have rectangular shape. Observed variables are shaded in gray. Boxes encircle the range of indices.

The full probabilistic model is sketched also in Fig 5, using the conventions of [64]. Reasoning types *τ*_*i*_ are sampled from a categorical distribution with a population-level *type distribution* *P*^*τ*^. In other words, the model assumes that there is a distribution of reasoning types from which our sample of participants was drawn. *A priori* the heterogeneous model *M*_*het*_ considers any type distribution *P*^*τ*^ equally likely, so that we sample it from a Dirichlet distribution with uniform weight 1 for all dimensions. That means that *M*_*hom*_ is nested under *M*_*het*_ as the special case where *P*^*τ*^ = 〈0, 1, 0〉 for the speaker population and *P*^*τ*^ = 〈0, 0, 1〉 for the listener population.

### Posteriors over model parameters

To learn about *a posteriori* credible values of parameters we used JAGS [65] to collect 10000 samples from the joint posterior distribution after a burn in of 10000 samples, from two chains with a thinning factor of 2. This set-up ensured convergence to the stationary distribution, with the $\hat{R}$ value of all continuous variables below 1.1 [66].

**Fig 6:**
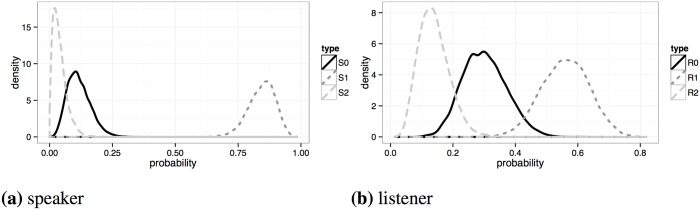
Marginal posterior for type priors *P^τ^* (*τ_i_*).

Summary statistics for the estimated marginal posteriors of model parameters are given in Table 3. Estimated marginal densities for the population priors are shown in Fig 6. The production data suggest that most speakers are *S*_1_ Gricean maximizers of relevant information, but also attest a small proportion of literal *S*_0_ speakers. In contrast, the data appear to give little support to hyper-pragmatic *S*_2_ speakers. These results are roughly in line with the homogeneous model, which assumes that speakers are of type *S*_1_. In contrast, the comprehension data give non-negligible levels of posterior credence to all three listener types. Interestingly, the Gricean listeners *R*_2_ are less likely than exhaustive listeners *R*_1_, and possibly even less likely than literal listeners *R*_0_. This seems to contradict the idea of *R*_2_-homogeneity in the population quite clearly, but proper model comparison is required to factor in model complexity as well (see below).

**Table 3:** 95% highest density intervals and means of marginal posteriors.

	speaker			listener		
	HDI min	mean	HDI max	HDI min	mean	HDI max
*ϵ*	9.5e-3	0.012	0.01498	1.88e-16	2.43e-4	1.11e-3
*λ*	2.956	3.330	3.711	5.424	5.856	6.298
*τ*_0_	0.036	0.117	0.209	0.173	0.301	0.442
*τ*_1_	0.741	0.843	0.940	0.410	0.560	0.703
*τ*_2_	8.43e-05	0.040	0.095	0.054	0.139	0.236

**Fig 7:**
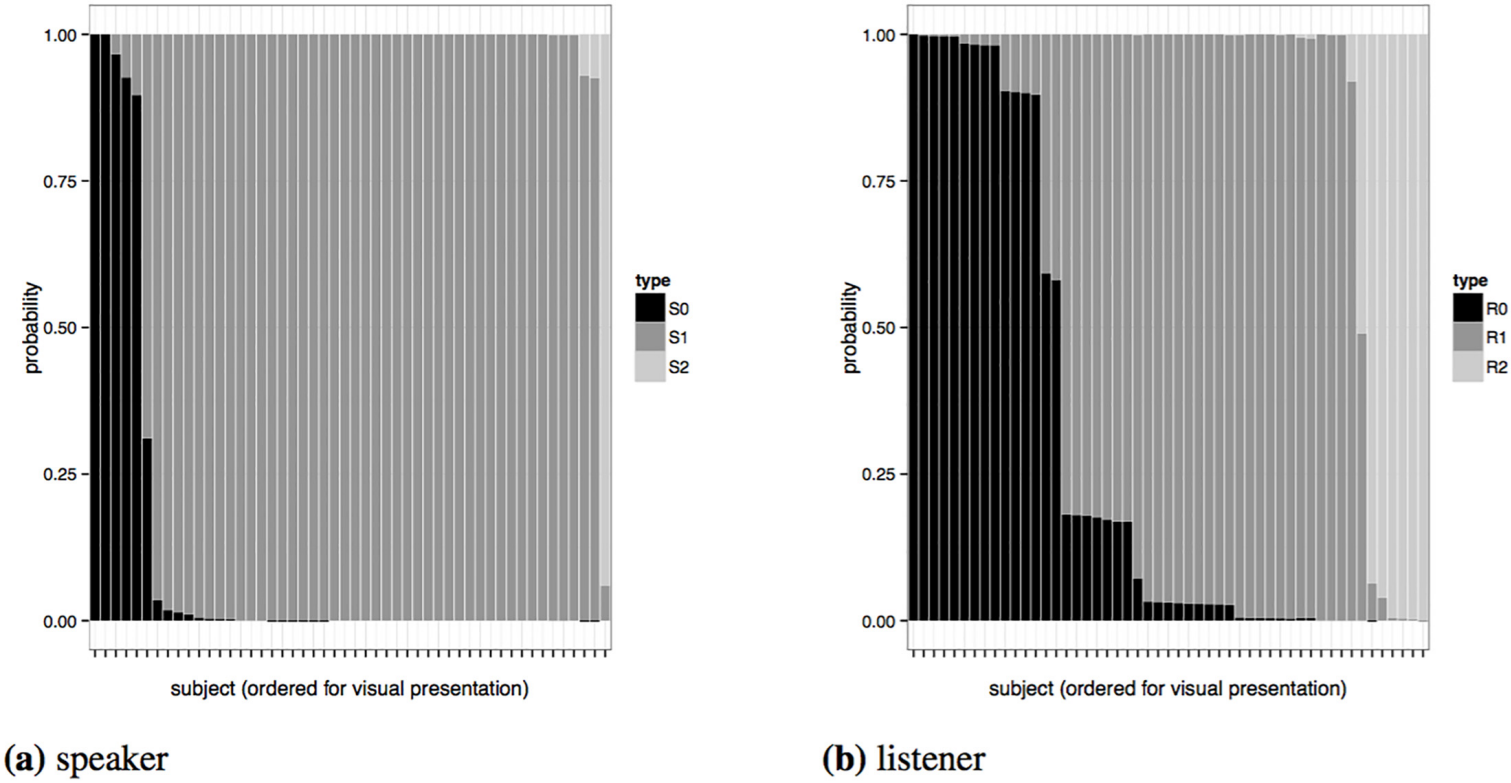
Marginal posterior for participants' reasoning types *τ_i_*.

Fig 7 shows the posterior distributions over participants’ reasoning types *τ*_*i*_. For production, most speakers’ data are best explained by assuming *S*_1_ behavior. There is only one participant that is most likely a hyper-pragmatic *S*_2_ speaker and there are four participants identified as most likely being literal *S*_0_ speakers. For most participants in the production experiment, our posterior beliefs in what type they should be, given the model and the data, have little uncertainty. In contrast, the comprehension data also identified most participants as almost certainly being of a single reasoning type, but there is somewhat more uncertainty in some cases. Nevertheless, there are only three participants whose classification by model and data yields ambiguous results. Interestingly, for all reasoning types, there are several participants that are clearly identified as most likely belonging to that type.

### Bayesian model comparison

When comparing models of different complexity, we need to weigh predictive accuracy against model parsimony. A Bayesian approach to model comparison does that [35, 36, 67–69]. Given our data *D*, we are interested in the ratio *P*(*M*_*hom*_∣*D*)/*P*(*M*_*het*_∣*D*) of posterior plausibility in favor of the simpler homogeneous model *M*_*hom*_ over the more complex heterogeneous model *M*_*het*_ given our data *D*. By Bayes’ rule this is the product of the ratio of the models’ *evidences* and the ratio of their prior probability:

$$\frac{P(M_{hom}|D)}{P(M_{het}|D)}=\frac{P(D|M_{hom})}{P(D|M_{het})}\times \frac{P(M_{hom})}{P(M_{het})}\;.$$

Irrespective of the strength of our prior beliefs in the models, the relative change in our belief brought about by the data is the *Bayes factor* *P*(*D* ∣ *M*_*hom*_)/*P*(*D* ∣ *M*_*het*_), which integrates over the predictions of the models under all its free parameter values, and thereby implements a bias against overfitting and model complexity:

$$\frac{P(D|M_{hom})}{P(D|M_{het})}=\frac{\int P\left(\theta |M_{hom}\right)\;P\left(D|\theta ,M_{hom}\right)\;d\theta }{\int P\left(\theta |M_{het}\right)\;P\left(D|\theta ,M_{het}\right)\;d\theta }\;.$$

Here, *θ* is a vector of values for all free model parameters. *P*(*θ* ∣ *M*_*hom*_) and *P*(θ ∣ *M_hom_*) are the priors over these under the respective models. The vector *θ* is the same for both models, but, as shown above, under the hierarchical approach taken here, the homogeneous model is *nested* under the heterogeneous model as the special case where *P*(*P*^*τ*^ = 〈0, 1, 0〉 ∣ *M*_*hom*_) = 1 for the speaker population and *P*(*P*^*τ*^ = 〈0, 0, 1〉 ∣ *M*_*hom*_) = 1 for the listener population.

Although the computation of Bayes factors can be difficult in general, there is an alternative, easier method for calculating Bayes factors of nested models, the *Savage-Dickey density-ratio* [34, 70]. Unfortunately, even with this method, precise calculation of Bayes factors is beyond reach for the complex models at hand. But approximation by simulation is possible. (Details and further explanations are provided in S5 Text.) To avoid precision problems, we look at the less extreme “null-hypotheses”: $P^{\tau }=\langle \frac{e}{2},1-e,\frac{e}{2}\rangle$ for production and $P^{\tau }=\langle \frac{e}{2},\frac{e}{2},1-e\rangle$ for comprehension. The higher we choose *e*, the more charitable we are to the homogeneous model (which is then no longer properly homogeneous, but still “approximately homogeneous”). With the fairly charitable value of 0.05, we receive Bayes factor approximations of:

$$\frac{P\left(D_{\text{prod}}|M_{hom}\right)}{P\left(D_{\text{prod}}|M_{het}\right)}\approx 3.955\;\frac{P\left(D_{\text{comp}}|M_{hom}\right)}{P\left(D_{\text{comp}}|M_{het}\right)}\approx 2.8e-11.$$

Bayes factors greater than 1 suggest that the data provide evidence in favor of the simpler homogeneous model. A standard recommendation is that only Bayes factors greater than 3 count as sufficiently strong evidence in favor of a model [34]. This means that under the charitable alternative “null-model” that says that exactly 95% of participants are Gricean *S*_1_ speakers, our data favor the simpler “almost only Gricean” model: its predictive success may be inferior, but due to factoring in model complexity, the posterior odds are shifted slightly towards it. However, if we lower our tolerance and test the assumption that exactly 99% of all participants are Gricean *S*_1_ speakers (*e* = 0.01), we obtain a Bayes factor of only 0.004 in favor of the Gricean model, i.e., very strong support for the more complex heterogeneous model (1/0.004 ≈ 250). In sum, the data support a moderate version of the idea that a large majority of speakers are Gricean, and even favors such a model over the full complexity and underspecification of the heterogeneous model, but our data do not support the idea that that majority is arbitrarily close to 100%.

On the other hand, even if we assume 95% Gricean *R*_2_ listeners, the odds are very clearly in favor of the complex heterogeneous model. This is also evident from the inferred most likely types of individual participants, shown in Fig 7: very few participants’ choice behavior can be best explained by the homogeneous model’s comprehension rule. What the Bayes factor analysis adds is the certainty that the heterogeneous model’s complexity is *necessary* for a decent explanation of the comprehension data; in other words, assuming that almost everybody is a Gricean listener is not a good explanation of the individual-level data.

Taken together, we reach the nuanced conclusion that the homogeneous Gricean model is half-supported by our data. Under a charitable interpretation, the idea that most (though not all) speakers are Gricean *S*_1_ speakers can be maintained, but not the idea that most listeners are Gricean *R*_2_ listeners. Instead, the data suggest that a majority of listeners are exhaustive *R*_1_ listeners. That is, there is variation in participants’ linguistic choices: a majority of participants use simple informativity-based heuristics, but a non-negligible number engages in deeper ToM-reasoning.

## General discussion

Recent years have seen a surge of success for probabilistic approaches to pragmatics, which have translated the Gricean picture of language use into a formal framework that makes quantitative, empirically testable predictions for speaker and listener behavior [7, 10, 12–14, 16–18, 20, 21]. In line with Grice’s original considerations, many of these approaches treat language users as Gricean speakers (*S*_1_) and Gricean listeners (*R*_2_). The models developed in this framework have provided a very good fit to aggregate population-level data across many different pragmatic tasks, suggesting that speakers and listeners are, on average, rational Gricean agents.

In contrast, individual-level data from our experiments on reference games suggest that it makes sense to believe that the population of participants is heterogeneous. While Gricean speakers and Gricean listeners are attested, the majority of participants seems to apply an information-based heuristic, corresponding to level-1 Theory-of-Mind reasoning: the majority of speakers are Gricean (*S*_1_), but the majority of listeners are exhaustive listeners (*R*_1_). This is despite the fact that a homogeneous Gricean population model does explain the population-level average reasonably well.

Although these results are contingent on the particular task we presented participants with, the obtained data, and the specific modeling choices we made, the demonstration that there can be discrepancies between the population- and individual-level perspective has at least two important implications for the growing field of probabilistic pragmatics, one more technical and one more conceptual. First, for probabilistic pragmatics to ripen, it is important in general to compare different relevant model variants stringently based on empirical data, especially when these variants are attested in the extant theoretical literature. Secondly, that individual differences in cognitive performance exist in many domains is beyond doubt, but the question arises whether a computational-level probabilistic pragmatics can accommodate these and whether it should care to do so. We will expand on these points in the following.

### Model variants & model comparison

Bayesian approaches to cognition have repeatedly been criticized as appearing conceptually under-motivated and as having insufficiently explored plausible alternatives [71–73]. Our contribution here is a partial response to these points of criticism, for we have pitted different variants of probabilistic pragmatic models against each other. In general, we believe that explicit model comparison is necessary in order to refine models and better understand what probabilistic pragmatics can and cannot achieve. Comparison of variants is especially relevant in cases like ours where the extant theoretical literature offers clearcut and plausible alternative modeling choices. When several model variants are conceivable, it can also be the case that these variants co-exist. In that case, hierarchical mixture models suggest themselves. We have tried to show here, by way of an example, how such hierarchical modeling could enrich the perspective of probabilistic pragmatics.

With the possibility of individual differences on the table, at least one possibility that we have not addressed so far is worth considering briefly. While we modeled individual differences in reasoning type, we assumed fixed population-level error parameters *λ* and *ϵ*. This is a simplifying assumption that served practical purposes, but it is important to note that individual-level variability in error parameters *alone* would not be a substitute for individual-level reasoning types. There is no single speaker or listener type (from the ones that we considered) that could capture the behavior of all other types as well, simply by choice of appropriate *λ*s and *ϵ*s. In this sense, individual variation in terms of reasoning types is not subsumed by individual variation in error levels. Moreover, although level-0 types can be emulated by level-(*n* ≥ 1)-types with *λ* close to 0, this is uninteresting as long as the research question is not whether a particular mathematical model can account for the data, but whether a particular conceptual idea can (e.g., literal interpretation vs. Gricean interpretation).

### Individual differences in probabilistic pragmatics

Probabilistic pragmatic models of the kind considered here are usually taken to provide computational-level analyses of pragmatic phenomena rather than algorithmic-level ones [74]. That is, the modeler specifies the problem that the agent needs to solve and provides a rational solution strategy, while making minimal assumptions about resource limitations [75]. Why should such a computational-level rational analysis care about individual differences?

First, if only aggregate data are considered, it is possible that the phenomenon we are trying to explain is an artifact of averaging [76]. If half the population is risk seeking and half is risk averse (to the same extent), then the average would indicate: the group is risk neutral. What would it then mean to explain risk neutrality as an optimal adaptation to functional pressure from the environment? In such an extreme case, a model that purportedly shows how the observed average behavior is rational need not be a model of anybody actually behaving rationally. This would be an odd instance of rational analysis: we would explain how adaptive pressure of the environment selected the group’s average behavior, not each individual’s behavior. Maybe such a line of explanation is possible, but in order to avoid these philosophical difficulties, inspection of individual-level data is key. If individual-level behavior aligns nicely with the average (modulo well-behaved noise) nothing is amiss.

If it does not, there are additional advantages to incorporating individual differences into computational-level models. One is bringing probabilistic pragmatics models closer to other processing-oriented models in psycholinguistics. Allowing for individual differences is one way of incorporating assumptions about varying degrees of resource limitations while remaining agnostic about what the algorithmic-level processes are that give rise to different computational-level player types. Nevertheless, one can speculate: plausible candidates are limitations on working memory, executive control, and other cognitive resources. In fact, there is a large body of literature identifying a role for these kinds of factors in language processing, theory of mind, and reasoning, which arguably constitute the intersection at which pragmatics lies. For example, speakers’ word order preferences are affected by working memory [77], differences in gesture production have been shown to be associated with individual differences in working memory as well as in spatial and verbal abilities [78, 79], working memory affects the rate at which prosody triggers contrastive inferences [80], and differences in perspective-taking have been shown to correlate with differences in inhibitory control [81]. In the reasoning literature, there is a large body of work showing that different cognitive biases (like the anchoring effect, belief bias, overconfidence bias, hindsight bias, base rate neglect, outcome bias, and sunk cost effect) are associated with differences in different types of intelligence, cognitive reflection, and openness (e.g., [82]). In this way, acknowledging individual differences and applying rational analysis at the individual level may help integrate computational-level probabilistic models with processing-oriented approaches.

In particular, when it comes to modeling language use, we believe that ignoring individual differences and focusing only on population-level data may hide that different sub-populations employ different production and comprehension strategies. This has been shown to play a role, for instance, in syntactic parsing, where ignoring individual differences can sometimes yield null effects when different subpopulations apply different parsing strategies [83]. Moreover, if the goal is to improve the predictive power of our cognitive models, what we have shown here is that taking into account individual differences is necessary for some cases of pragmatic reasoning. Whether it is necessary for all cases is an empirical question that should be addressed in future work. Relatedly, whether an individual’s inferred player type on one pragmatic task transfers to another pragmatic task is another interesting open question.

For all the above discussed reasons, an empirically adequate theory of pragmatic language use might ultimately like to predict individuals’ behavior, not just population-level means. A final illustration of this point comes from taking the perspective of a speaker engaged in dialog. Speakers tend to not speak into a vacuum, but instead interact with a particular interlocutor about whose behavior they may have rather concrete beliefs. When speaking with a complete stranger, speakers may start out with the assumption that their interlocutor is like the population mean. However, speakers and listeners immediately obtain information about their interlocutor’s identity that they condition on. Indeed, there is an increasing body of work showing that interlocutors rapidly adapt to each others’ phonetic [84], lexical [85], and syntactic preferences [86, 87]. One of the interlocutor’s attributes is arguably the amount of effort they can or are willing to invest in drawing inferences, or, put differently: their player type. If I know my interlocutor’s type, I can make more accurate predictions about their use of language, thereby increasing the chance of communicative success. This entails that interlocutors should track each others’ player type. Some evidence that they do comes from studies on speaker-specific overinformativeness, showing that listeners rapidly adapt to the level of informativeness of speakers’ use of adjectives [25, 26], but much more work is needed to investigate the extent to which interlocutors represent player type.

## Conclusion

In this paper we set out to answer a simple question: are listeners and speakers Gricean at the individual level or only at the population level? To test this, we inferred individually variable pragmatic reasoning types as latent parameters in a hierarchical model and used Bayesian model comparison to draw conclusions about whether the added complexity of maintaining the possibility of non-Gricean reasoning types is required by our data. Data came from one production and one comprehension study in reference games that required participants to apply pragmatic reasoning of varying complexity. While there was evidence for a substantial number of likely Gricean speakers and Gricean listeners, the added complexity of considering additional reasoning types was justified, especially in comprehension, where many listeners were identified as exhaustifiers. In conclusion, given our data for this task and our model, there are clear individual differences between participants; for the case of Quantity inferences in referential expressions, most speakers and listeners seem to apply an information-based heuristic, corresponding to level-1 ToM-reasoning. This suggests that probabilistic pragmatics models should take into account potential individual variation, even if designed as a computational-level theory, and this paper demonstrates one way of doing so.

## Supporting Information

S1 Text Derivation of Predictions for Idealized Reasoning Types. (PDF)

S2 Text Explanation of Heterogeneous Type Definitions. (PDF)

S3 Text URLs for Experiments. (PDF)

S4 Text Details on the Prior Elicitation Experiment. (PDF)

S5 Text Details on the Calculation of Bayes Factors. (PDF)

S6 Text Posterior Predictive Check for Heterogeneous Model. (PDF)

S1 Data Data from Experiment 1. (CSV)

S2 Data Data from Experiment 2. (CSV)
