# Supplementary material for: Reasoning in Reference Games: Individual- vs. Population-Level Probabilistic Modeling
Source: PLoS One. 2016 May 5;11(5):e0154854. doi: 10.1371/journal.pone.0154854 (PMC4858259; doi:10.1371/journal.pone.0154854)
Supplement: S1 Text — (PDF) [file pone.0154854.s001.pdf]

Supplementary Information 1

Reasoning in Reference Games: Individual- vs. Population-Level Probabilistic Modeling

Choice Behavior of Idealized Reasoning Types

The behavioral predictions of (idealized, non-probabilistic) reasoning types for the simple and complex conditions in Fig. 1 of the main text are plotted schematically in Fig. 8. There are two strands of reasoning types, one starting with a literal interpreter (R_0, the upper rows), and one starting with a literal speaker (S_0, the lower rows). Each of the four diagrams should be read from left to right. Interpretation behavior is a function from utterances (denoting properties) to referents; production behavior is a function from referents to utterances (denoting properties). These functions are represented here by left-to-right arrows.

To see how these diagrams capture rational reasoning in reference games, consider the upper row of Fig. 8a first. A literal listener R_0 would interpret "red hat" as referring equally likely to the green monster with the red hat and the robot with the red hat. The Gricean speaker S_1 would, however, choose the description "green monster" for the green monster (because that gives him a probability 1 chance of inducing the right interpretation given his belief that the listener interprets literally, as compared to a probability of .5 when signaling "red hat"). The Gricean interpreter R_2 would interpret "red hat" to refer to the robot with the red hat, because that is the only referent for which the Gricean speaker would use that description (recall that in this setup there is no message corresponding to "robot"). Higher level types would simply repeat the behavior of S_1 and R_2; the sequence has reached a fixed point. In sum, Gricean speakers and listeners "solve" the simple condition in the sense that they would prefer the target option over the competitor. But even exhaustive R_1 interpreters can "solve" the simple condition. R_1, who reasons about what an unbiased literal speaker would say, can conclude that it is twice as likely that the speaker refers to the target (robot) than that she refers to the competitor (green monster). This is because there is only one true description for the robot, whereas there are two for the green monster. Consequently, all types of at least level 1 can solve the simple condition. Similar arguments show that only level 2 agents can solve the complex condition in Fig. 1b, as pictured in Fig. 8b (see Degen and Franke, 2012; Degen et al., 2013). More concretely, going case by case:

- In *simple production trials*, the designated referent is the green monster with the red hat. It is indicated by an asterisk in Fig. 1a. The speaker's choice options are the four pictorial messages on the right in Fig. 1a. Only the target message ("green monster") and the competitor ("red hat") are true; the other two messages we call distractors. The former would be selected with equal likelihood by a hypothetical literal sender S_0. However, a Gricean speaker S_1 would choose the target message "green monster", because that would be an unambiguous description, unlike the competitor "red hat." This is what an S_1 speaker would do, according to the idealized IBR types. So, both S_1 and S_2 can solve the simple condition.

- In *simple comprehension trials*, the listener is to interpret the description "red hat," as indicated by an asterisk in Fig. 1a. There are two interpretations for which that messages is true: the target interpretation (robot) and the competitor interpretation (green monster). The literal interpreter R_0 would choose either with equal likelihood, but exclude the distractor interpretation (purple monster). But an exhaustive interpreter R_1, who reasons about what an unbiased literal speaker would say, can conclude that it is twice as likely that the speaker refers to the target (robot) than that she refers to the competitor (green monster). This because there is only one true description for the robot, whereas there are two for the green monster. Consequently, both R_1 and R_2 can solve the simple condition.

- In *complex production trials*, the designated referent is the green monster with the red hat, indicated by an asterisk in the Fig. 1b. Now, both the target message "red hat" and the competitor message "green monster" are two-way ambiguous under their obvious semantic interpretation. Consequently, even a Gricean speaker S_1

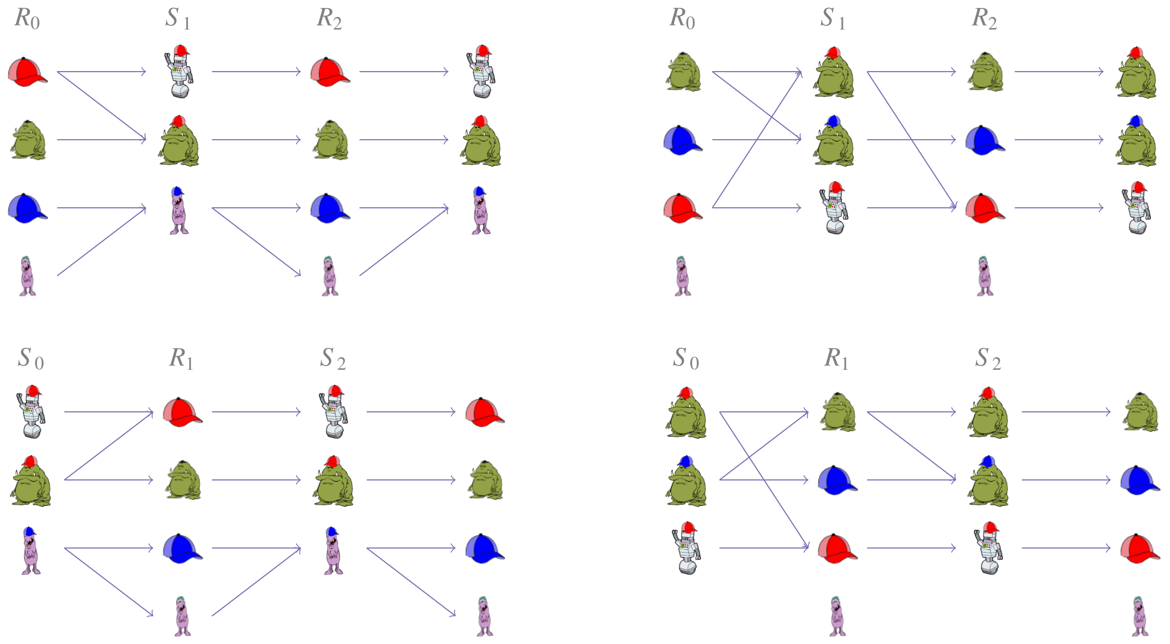

(a) simple (b) complex

Figure 8: IBR reasoning applied to the simple and complex reference games from Fig. 1.

would be indifferent between them, since they are equally informative under a literal interpretation. But hyper-pragmatic S_2 speakers assume that an R_1 listener would only interpret the target message "green monster" as possibly referring to the designated referent. This is because the competitor message "red hat" is the only description usable for the robot. So, by taking R_1's pragmatically strengthened interpretations into account, S_2 would pick the target message "green monster." In sum, only S_2 solves the complex condition.

- In *complex comprehension trials*, the listener is to interpret the message " green monster." This is semantically ambiguous in the context between the target referent (green monster with a red hat) and the competitor (green monster with the blue hat). It is therefore equally likely to be sent by a literal speaker. So, both R_0 and R_1 would choose the target and the competitor referent with equal probability. But a Gricean interpreter R_2 would assume that a Gricean speaker S_1 would describe the green monster with the blue hat with the unambiguous description "blue hat." Hence, R_2 would pick the target referent in this case.

References

Degen, Judith and Michael Franke (2012). "Optimal Reasoning About Referential Expressions". In: *Proceedings of SemDial 2012 (SeineDial): The 16th Workshop on the Semantics and Pragmatics of Dialogue*. Ed. by Sarah Brown-Schmidt, Jonathan Ginzburg, and Staffan Larsson, pp. 2–11.

Degen, Judith, Michael Franke, and Gerhard Jäger (2013). "Cost-Based Pragmatic Inference about Referential Expressions". In: *Proceedings of the 35th Annual Meeting of the Cognitive Science Society*. Ed. by Markus Knauff, Michael Pauen, Natalie Sebanz, and Ipke Wachsmuth. Austin, TX: Cognitive Science Society, pp. 376–381.
