# Supplementary material for: Reasoning in Reference Games: Individual- vs. Population-Level Probabilistic Modeling
Source: PLoS One. 2016 May 5;11(5):e0154854. doi: 10.1371/journal.pone.0154854 (PMC4858259; doi:10.1371/journal.pone.0154854)
Supplement: S2 Text — (PDF) [file pone.0154854.s002.pdf]

Supplementary Information 2

Reasoning in Reference Games: Individual- vs. Population-Level Probabilistic Modeling

Heterogenous Type Model

While the RSA model features a literal interpreter R_0 only as a technical dummy construct, we here include the possibility that some listeners actually only interpret utterances literally. Likewise, we include the possibility that speakers are just producing literally true descriptions without any further pragmatic considerations. To further allow for the possibility that literal speakers and listeners make mistakes, we define them as agents who soft-maximize a utility measure that implements compliance with semantic meaning only (c.f. Degen et al., 2013; Franke and Jäger, 2014):

$$S_0(m \mid t; \lambda) \propto \exp(\lambda\, \mathcal{U}(m \mid \{m' \mid m' \text{ is true of } t\}))$$
$$R_0(t \mid m; \lambda) \propto \exp(\lambda\, \mathcal{U}(t \mid \{t' \mid m \text{ is true of } t'\}))\,.$$

Higher-order speaker types are defined, in keeping with the RSA framework:

$$S_{n+1}(m \mid t; \lambda, \epsilon) \propto S'_{n+1}(m \mid t; \lambda) + \epsilon$$
$$\text{with} \quad S'_{n+1}(m \mid t; \lambda) \propto \exp(\lambda \cdot \log R_n(t \mid m))\,,$$

where $R_n(t \mid m) = \lim_{\lambda \to \infty} R_n(t \mid m; \lambda)$ is the noise-free, purely rational behavior of a level-n listener. This is to avoid nesting of λ-s for one agent's beliefs about the rationality of another (see Qing and Franke, 2015 for comparison of models that do and do not allow for this).

As for production, we will consider only the first three speaker types S_0, S_1, and S_2. This is because higher level types qualitatively match the behavior of S_2 in all relevant conditions of our experiment. The patterns of behavior captured by these types are sketched in Fig. 9 for different λ-values. The literal speaker S_0 will have equal choice probability for target and competitor in both simple and complex conditions. The Gricean speaker S_1 of the RSA model will have high probabilities for choosing the target message in the simple condition for sufficiently high values of λ, but will have equal probability of choosing target and competitor messages in the complex condition. Finally, the more sophisticated hyper-pragmatic S_2 players have high target choice probabilities for both simple and complex conditions.

As for comprehension, we would like to retain R_2 exactly as defined in the RSA model. In parallel to the above pattern of speaker types, we would like to have an additional listener type that can neither solve the simple nor complex condition (R_0) and one that can solve the simple, but not the complex condition (R_1). We therefore define R_1 as:

$$R_1(t \mid m, \lambda) \propto \exp(\lambda\ \mathrm{U}_{R_1}(t, m))$$
$$\text{with} \quad \mathrm{U}_{R_1}(t, m) \propto \mathcal{U}(t) \cdot S_0(m \mid t)\,,$$

where, again, $S_0(m \mid t) = \lim_{\lambda \to \infty} S_0(m \mid t; \lambda)$ is a noise-free literal speaker.

Two remarks are in order in justification of the definition of R_1. Firstly, we use a uniform prior over referents $\mathcal{U}(t)$, not the empirically measured salience prior $\mathcal{S}(t)$. This is because we think of R_1 in parallel to S_1 as a listener that implements an interpretation heuristic, rather than strict contextualized pragmatic reasoning. This way, R_1 implements (a probabilistic approximation of a version of) exhaustive interpretation (Franke, 2011), and so implements an approach to pragmatic interpretation that is prominent in theoretical linguistics. Secondly, although the alternative definition $R_1(t \mid m, \lambda_1) \propto \mathcal{U}(t) \cdot S_0(m \mid t, \lambda)$ would have been superficially closer to RSA's listener rule, it would not be a listener type that solves the simple condition, but would approach a maximal target choice probability of only $\frac{2}{3}$.

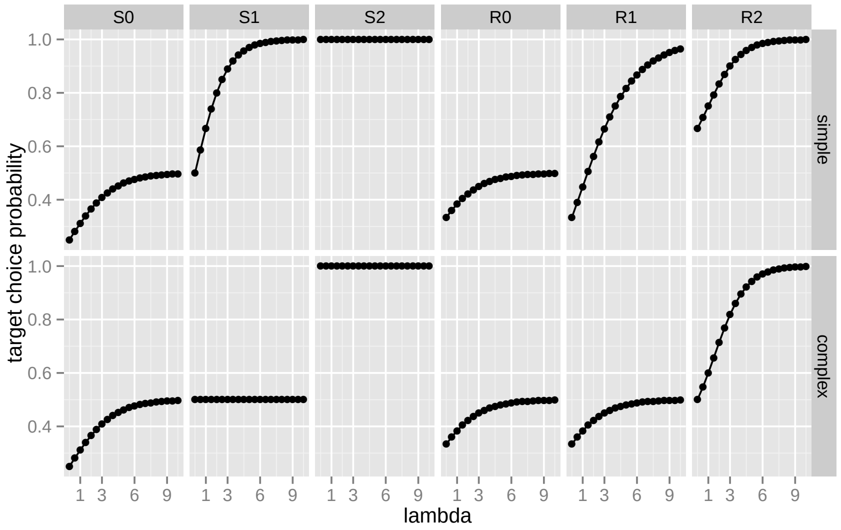

Figure 9: Predictions of the probability of choosing the target (referent or message) in the simple and complex condition for the listener and speaker types defined in the heterogeneous model, for different values of rationality parameter λ and $\epsilon = 0$, under the assumption that all (salience) priors are flat.

References

Degen, Judith, Michael Franke, and Gerhard Jäger (2013). "Cost-Based Pragmatic Inference about Referential Expressions". In: *Proceedings of the 35th Annual Meeting of the Cognitive Science Society*. Ed. by Markus Knauff, Michael Pauen, Natalie Sebanz, and Ipke Wachsmuth. Austin, TX: Cognitive Science Society, pp. 376–381.

Franke, Michael (2011). "Quantity Implicatures, Exhaustive Interpretation, and Rational Conversation". In: *Semantics & Pragmatics* 4.1, pp. 1–82.

Franke, Michael and Gerhard Jäger (2014). "Pragmatic Back-and-Forth Reasoning". In: *Semantics, Pragmatics and the Case of Scalar Implicatures*. Ed. by Salvatore Pistoia Reda. Palgrave Studies in Pragmatics Language and Cognition. New York: Palgrave MacMillan. Chap. 7, pp. 170–200.

Qing, Ciyang and Michael Franke (2015). "Variations on a Bayesian Theme: Comparing Bayesian Models of Referential Reasoning". In: *Bayesian Natural Language Semantics and Pragmatics*. Ed. by Henk Zeevat and Hans-Christian Schmitz. Language, Cognition and Mind. Berlin: Springer, pp. 201–220.
