# Supplementary material for: Reasoning in Reference Games: Individual- vs. Population-Level Probabilistic Modeling
Source: PLoS One. 2016 May 5;11(5):e0154854. doi: 10.1371/journal.pone.0154854 (PMC4858259; doi:10.1371/journal.pone.0154854)
Supplement: S3 Text — (PDF) [file pone.0154854.s003.pdf]

Supplementary Information 3

Reasoning in Reference Games: Individual- vs. Population-Level Probabilistic Modeling

URLs of experiments

All experiments reported in this paper can be viewed online at the following URLs:
Experiment 1: `http://stanford.edu/~jdegen/59_monsterscales/monsters.html`
Experiment 2: `http://stanford.edu/~jdegen/61_monsterscales_production/monsters.html`
Experiment 3: `http://web.stanford.edu/~jdegen/62_monsterscales_prior/monsters.html`
