# Supplementary material for: Reasoning in Reference Games: Individual- vs. Population-Level Probabilistic Modeling
Source: PLoS One. 2016 May 5;11(5):e0154854. doi: 10.1371/journal.pone.0154854 (PMC4858259; doi:10.1371/journal.pone.0154854)
Supplement: S4 Text — (PDF) [file pone.0154854.s004.pdf]

# Supplementary Information 4

Reasoning in Reference Games: Individual- vs. Population-Level Probabilistic Modeling

## Experiment 3: prior elicitation

In order to evaluate higher-level listener behavior, it was necessary to obtain estimates of the prior salience of each of the three creatures in the display for each display used in Experiment 1. Experiment 3 was conducted to collect these estimates.

**Participants.** 129 participants were recruited via Amazon's Web Service Mechanical Turk. Participants' IP address was limited to US addresses only. Only participants with a past work approval rate of at least 95% were accepted.

**Ethics statement.** This study was conducted with the approval of the Stanford University research subjects review board. All participants gave written consent and received $0.30 for their participation (hourly rate of $10.00) according to the policies set forth by the Stanford University research subjects review board.

**Procedure and materials.** Trials were similar to comprehension trials in Experiment 1. On each trial, participants chose one object from a triple of objects. But instead of receiving a message, participants were instead told (following Frank and Goodman, 2012):

> Imagine someone is talking to you in an alien language and uses a word you don't know to refer to one of these creatures. **Which creature do you think the person is talking about?** Click on that creature.

Triples and their spatial arrangement were randomly sampled from the 157 different visual displays (grids) of referents that occurred in Experiment 1. Each participant completed 26 experimental trials. Thus, each unique grid was was seen between participants on average 21 times. Each grid was seen at least 16 times and no grid was seen more than 31 times.

**Results.** From the obtained choice distributions over objects in each grid, salience priors were computed for each of the four reference game types that we considered: simple, complex, unambiguous and ambiguous. Since the same grid appeared in different conditions in Experiment 1, we determined for each condition the set of grids that occurred with that condition in Experiment 1, together with its occurrence frequency for that condition. The salience priors for each condition are then the average of the choices from Experiment 3 weighted by the frequency of occurring with the relevant condition in Experiment 1. The resulting salience priors are given in Table 4.

Table 4: Results from the salience prior elicitation in Experiment 3.

| | target | competitor | distractor |
|---|---|---|---|
| simple | .340 | .253 | .407 |
| complex | .221 | .380 | .399 |
| unambiguous | .280 | .203 | .518 |
| ambiguous | .119 | .121 | .760 |

## References

Frank, Michael C. and Noah D. Goodman (2012). “Predicting Pragmatic Reasoning in Language Games”. In: *Science* 336.6084, p. 998.
