# Supplementary material for: Reasoning in Reference Games: Individual- vs. Population-Level Probabilistic Modeling
Source: PLoS One. 2016 May 5;11(5):e0154854. doi: 10.1371/journal.pone.0154854 (PMC4858259; doi:10.1371/journal.pone.0154854)
Supplement: S5 Text — (PDF) [file pone.0154854.s005.pdf]

Supplementary Information 5

Reasoning in Reference Games: Individual- vs. Population-Level Probabilistic Modeling

Bayes factor approximations

The Savage-Dickey density ratio (Dickey and Lientz, 1970; Wagenmakers et al., 2010) tells us that if the nested model M_{hom} fixes the value $\theta_1 = v_1$, then the Bayes factor of the nested model comparison is given by the ratio of, roughly put, how much the data change our belief in this value, from the point of view of the complex model:[1]

$$\frac{P(D \mid M_{hom})}{P(D \mid M_{het})} = \frac{P(\theta_1 = v_1 \mid D, M_{het})}{P(\theta_1 = v_1 \mid M_{het})} \,. \tag{1}$$

Intuitively, if the data make it appear less likely that $\theta_1 = v_1$ than assumed *a priori*, then this is evidence for the more complex model; conversely, if the posterior level of credence in $\theta_1 = v_1$ goes up, it appears that the simpler model got it right and should be preferred. At the same time, we see the bias against overfitting and model complexity: the more parameter values the complex model considers *a priori* likely for θ_1, the lower will be the prior density for $\theta_1 = v_1$, and so the more the simple model will be favored if the belief in $\theta_1 = v_1$ is not sufficiently strongly undermined by the data.

To measure the extent to which our data favor the simple model M_{hom} over the more complex M_{het} using the Savage-Dickey density ratio, we need to calculate the ratio given by Equation (1), which we can make more precise, e.g., for the production data:

$$\frac{P(D_{\text{prod}} \mid M_{hom})}{P(D_{\text{prod}} \mid M_{het})} = \frac{P(P^\tau = \langle 0,1,0 \rangle \mid D_{\text{prod}}, M_{het})}{P(P^\tau = \langle 0,1,0 \rangle \mid M_{het})} \,.$$

In words, the Bayes factor in favor of M_{hom} has as its numerator the marginal posterior density, given the data and the complex model M_{het}, of a type distribution that excludes literal and hyper-pragmatic speakers. The denominator is the prior probability of that type distribution in the complex model.

The latter can easily be calculated. In M_{het}, prior values of P^τ come from an unbiased Dirichlet distribution with a weight vector $\alpha = \langle 1,1,1 \rangle$. So every value of P^τ has equal density, i.e., by definition of the Dirichlet distribution:

$$P(P^\tau = \langle x_1, x_2, x_3 \rangle \mid M_{het}) = \frac{\Gamma(\sum_{i=1}^{3} \alpha_i)}{\prod_{i=1}^{3} \Gamma(\alpha_i)} \prod_{i=1}^{3} x_i^{\alpha_i - 1} = \frac{2}{1} \, 1 = 2 \,.$$

So, the denominator of the Bayes factor will be 2 for both production and comprehension.

The numerators cannot (easily) be computed analytically. But we can use the samples of our MCMC runs to estimate the posterior density. We take a parametric approach here. Concretely, we assume that the "true" posterior for values P^τ is a Dirichlet distribution, $P(P^\tau \mid D, M_{het}) = \text{Dirichlet}(P^\tau; \langle \alpha_1, \alpha_2, \alpha_3 \rangle)$, with unknown Dirichlet weights $\langle \alpha_1, \alpha_2, \alpha_3 \rangle$. The MCMC sample values of P^τ then let us estimate these weights, using a maximum likelihood approach. The best-fitting weights, given our sample data, are shown in Table 5.[2]

[1] This result holds in general, as long as the priors of the complex model satisfy the continuity condition $\lim_{\theta_1 \to v_1} P(\theta_2, \ldots, \theta_n \mid M_{het}, \theta_1) = P(\theta_2, \ldots, \theta_n \mid M_{hom})$. This condition is met in our case, because the prior over type distributions $P(P^\tau \mid M_{het})$, to which M_{hom} assigns a fixed value, is independent of all other model parameters.

[2] To validate the parametric approach, i.e., to check whether it is plausible that our samples of P^τ could be the result of sampling from a Dirichlet distribution with the best-fitting weights, we generated another 1000 samples of 10000 probability distributions from the Dirichlets with the best-fitting weights. Each of these 1000 samples (of 10000 probability distributions) is then like our 1 MCMC sample of P^τ-values. We looked at the likelihoods of each of these former samples under the generating best-fit Dirichlets, and computed the 95% HDIs over those likelihoods. This gives us an expected range of likelihoods that samples from the allegedly true distributions would show. We then checked whether the likelihood of the

Table 5: Maximum-likelihood fit of Dirichlet posterior over P^τ.

	MLE-fitted values			simulation comparison
	α_1	α_2	α_3	95% HDI percentile
speaker	5.392	39.009	1.837	49.30%
listener	13.508	25.116	6.272	51.46%

The numerators of the relevant Bayes factors can then be approximated by the density of the point-values of interest under the Dirichlet distributions with the respective best-fitting weights. The problem here is that the extreme assumptions of the homogeneous model, $P^\tau = \langle 0, 1, 0 \rangle$ for production and $P^\tau = \langle 0, 0, 1 \rangle$ for comprehension, receive probability zero, because the best-fitting weights are all bigger than one. For a fairer comparison, we therefore look at the less extreme "null-hypotheses": $P^\tau = \left\langle \frac{e}{2}, 1 - e, \frac{e}{2} \right\rangle$ for production and $P^\tau = \left\langle \frac{e}{2}, \frac{e}{2}, 1 - e \right\rangle$ for comprehension (see main text for results).

References

Dickey, James M. and B. P. Lientz (1970). "The Weighted Likelihood Ratio, Sharp Hypotheses about Chances, the Order of a Markov Chain". In: *The Annals of Mathematical Statistics* 41.1, pp. 214–226.

Wagenmakers, Eric-Jan, Tom Lodewyckx, Himanshu Kuriyal, and Raoul Grasman (2010). "Bayesian hypothesis testing for psychologists: A tutorial on the Savage–Dickey method". In: *Cognitive Psychology* 60, pp. 158–189.

MCMC samples was within that credible range of likelihoods. For both production and comprehension cases, it was. Table 5 lists the percentile of where the likelihood of the original MCMC samples lie on the 95% HDIs of the likelihoods of synthetic samples. This suggests that our parametric approach is warranted: the MCMC samples of P^τ "look" very much like what we would expect under equally-sized samples from Dirichlets with the best-fitting weights.
