# Supplementary material for: Reasoning in Reference Games: Individual- vs. Population-Level Probabilistic Modeling
Source: PLoS One. 2016 May 5;11(5):e0154854. doi: 10.1371/journal.pone.0154854 (PMC4858259; doi:10.1371/journal.pone.0154854)
Supplement: S6 Text — (PDF) [file pone.0154854.s006.pdf]

Supplementary Information 6

Reasoning in Reference Games: Individual- vs. Population-Level Probabilistic Modeling

Posterior Predictive Check

Model comparison only provides relative information: one model can be deemed better than another. This does not yet tell us whether the better model is good enough in absolute terms. To check whether the heterogeneous model is able to capture the data reasonably well, a *posterior predictive check* helps: we sample 10000 values of parameters from the estimated joint posterior distribution and generate a prediction of a potential observation for each of these; if the observed data are unexpected, given the posterior predictive samples, we should consider the possibility that the model misses crucial patterns in the data. We consider a posterior predictive check to the population-level target choice frequencies, so that we get a comparable assessment to the homogeneous model's performance reported in the main text.

Fig. 10 gives the estimated densities of the posterior predictive, together with their 95% HDIs and an indication of the observed number of counts. The observations all lie in the 95% HDIs, so that we may conclude that the heterogeneous model, if trained on the individual-level data, is no longer "surprised" by the population-level data. This, then, is a very rudimentary sanity check, showing that the heterogeneous model, if conditioned on the individual-level data, captures the general patterns in the population-level data as well.

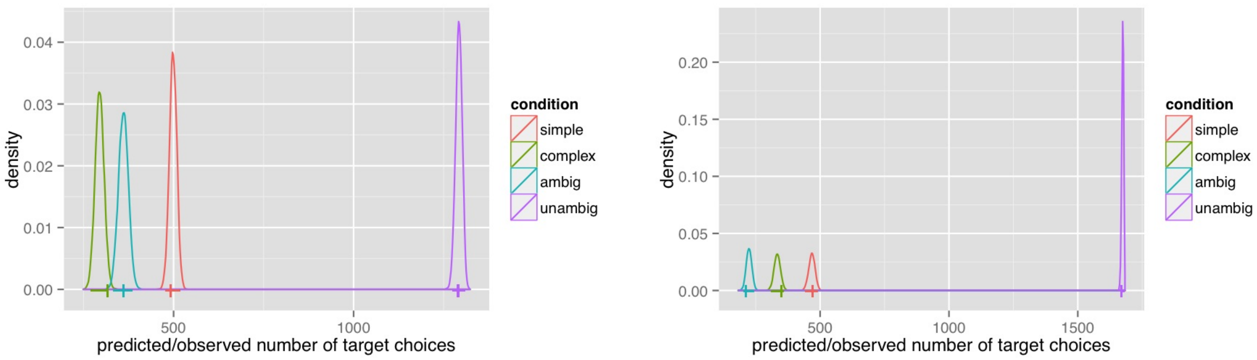

(a) speaker

(b) listener

Figure 10: Posterior predictive checks: density estimates for the total number of target choices predicted under the posterior distribution over parameter values. Under each estimated density, the horizontal colored lines indicate the respective 95% HDIs. The vertical bars mark the empirically observed total counts of target choices. All observed counts lie within the relevant 95% HDIs.

In sum, this suggests that the heterogeneous model can compete with the homogeneous model when it comes to predicting the population-level data, although considerations of parsimony would in this case certainly favor the simpler homogeneous model. But the heterogeneous model very clearly has the upper hand when it comes to predicting individual-level data. It seems that, in allusion to the "wisdom of the crowds" (Surowiecki, 2004), there is good reason to believe in "Grice-dom of the crowds," but not in "Grice-dom of each individual," at least as far as reference games and the data from the reported experiments are concerned.

References

Surowiecki, James (2004). *The Wisdom of the Crowds: Why the Many are Smarter than the Few and How Collective Wisdom Shapes Business, Economies, Societies and Nations*. Anchor Books.
